# Warming Houses

**Published:** 1875-01

**Authors:** 


WARMING HOUSES.

This is an important topic, as our numerous letters fully attest. The thoughtful portion of the public want the best heating device, and they want to combine reasonable economy with the highest sanitary conditions. They do not wish to breathe bad air, simply because it becomes a necessity that they should breathe heated air. They are not willing to destroy themselves and their families through the heating apparatus.

We are convinced that Mr. A. M. Lesley's Gothic Furnace, to be found at 224 and 226 W. Twenty-third street, is the best we have examined. We have never used it, or lived in a house heated by it, but we have much valuable testimony to its value. The last gentleman with whom we conversed on the subject was R. G. Beardslee, Esq., a member of the Board of Education of New York, and a prominent lawyer. He gave us a very interesting account of his experience with furnaces in his house, No. 47 West Fifty-fifth street. He had a popular furnace, which was considered all right, but he found some leakage of gas and other unsatisfactory effects. He concluded that he must have a new heating device, and he began to make inquiries. Mr. Douglass, who owns and occupies the fine residence, corner of Forty-first street and Fifth avenue, advised him to try the "Gothic." Investigation satisfied him that he could find no more efficient and economical heater than this. So he bought a Number 12—the largest size. He selected this size because Mr. Douglass had the same. But there was an objection to it. It kept his house uncomfortably and unwholesomely hot. Why it should do so he could not comprehend. But he reflected that Mr. Douglass' house was on a corner while his own house was in the centre of a block, and so protected from wind. But the heat was so excessive that he decided to change the furnace. He talked with Judge Bosworth, who was using a Number 10 Gothic, and was advised by the Judge to try that size. Accordingly he got Mr. Lesley to put in a Number 10 in place of the Number 12, and the result was eminently satisfactory. Judge Bosworth had told him that his Number 10 heated his large house through the winter with four tons of coal. Mr. Beardslee hardly equals this, but he speaks in very high terms of the Gothic, of its heating power, and of the wholesome quality of the warm air which it supplies. Some of our friends who are about to build have looked at the various furnaces extant, and decide to put in the "Gothic."

---

STUTTERING CURED.—"The Judge Walworth has boiled his buster!" cried out the alarmed horseman, as he galloped into New Haven, to announce the blowing up of a steamboat. This was a form of stuttering; the mind went ahead of the ability of rapid utterance.

All stutterers are of the nervous temperament, and in their haste to speak, so much nervous energy is sent to the vocal organs that they cannot act fast enough for it to escape, and confusion is the result. All that is necessary is to divert a part of that energy to some other operation; that is, to divert the mind partly to some other thing; thus, at the enunciation of every syllable tap any part of the body with the finger, or the floor with the foot, or wink the eye, and the person will cease to stutter on the instant. Persist in the practice and the cure will be permanent.

---

Bathe very quickly in winter.

